# Review of Big Data Analytics, Artificial Intelligence and Nature-Inspired Computing Models towards Accurate Detection of COVID-19 Pandemic Cases and Contact Tracing

**DOI:** 10.3390/ijerph17155330

**Published:** 2020-07-24

**Authors:** Israel Edem Agbehadji, Bankole Osita Awuzie, Alfred Beati Ngowi, Richard C. Millham

**Affiliations:** 1 Office of the Deputy Vice Chancellor: Research, Innovation and Engagement, Central University of Technology, Bloemfontein 9301, South Africa; angowi@cut.ac.za; 2 Centre for Sustainable Smart Cities 4.0, Faculty of Engineering, Built Environment and Information Technology, Central University of Technology, Bloemfontein 9301, South Africa; bawuzie@cut.ac.za; 3 ICT and Society Research Group, Department of Information Technology, Durban University of Technology, Durban 4001, South Africa; richardm1@dut.ac.za

**Keywords:** contact tracing, 2019 novel coronavirus disease (COVID-19), nature-inspired computing (NIC), artificial intelligence (AI), big data

## Abstract

The emergence of the 2019 novel coronavirus (COVID-19) which was declared a pandemic has spread to 210 countries worldwide. It has had a significant impact on health systems and economic, educational and social facets of contemporary society. As the rate of transmission increases, various collaborative approaches among stakeholders to develop innovative means of screening, detecting and diagnosing COVID-19’s cases among human beings at a commensurate rate have evolved. Further, the utility of computing models associated with the fourth industrial revolution technologies in achieving the desired feat has been highlighted. However, there is a gap in terms of the accuracy of detection and prediction of COVID-19 cases and tracing contacts of infected persons. This paper presents a review of computing models that can be adopted to enhance the performance of detecting and predicting the COVID-19 pandemic cases. We focus on big data, artificial intelligence (AI) and nature-inspired computing (NIC) models that can be adopted in the current pandemic. The review suggested that artificial intelligence models have been used for the case detection of COVID-19. Similarly, big data platforms have also been applied for tracing contacts. However, the nature-inspired computing (NIC) models that have demonstrated good performance in feature selection of medical issues are yet to be explored for case detection and tracing of contacts in the current COVID-19 pandemic. This study holds salient implications for practitioners and researchers alike as it elucidates the potentials of NIC in the accurate detection of pandemic cases and optimized contact tracing.

## 1. Introduction

Currently, the COVID-19 pandemic which was first reported in Wuhan, which is situated within China’s Hubei province, in December 2019 has affected, at the time of writing this article, 213 countries with confirmed cases totalling 10,614,903 recovered cases totalling 5,824,883 and death cases standing at an estimated 514,626 [1]. This has culminated in the labelling of the disease as a pandemic by the World Health Organization (WHO). COVID-19 is being transmitted through human beings at an alarmingly incredible pace and consequently, it has affected the economies of several countries grounding the healthcare, education and transportation sectors, among others. The disease is highly contagious, and it is contracted through respiratory droplets originating from an infected person. The mild cases of COVID-19 have such symptoms as shortness of breath, fever, sputum production and muscle pain, whereas severe cases may lead to pneumonia and multi-organ failure [2]. Currently, there are no vaccines clinically proven for treating COVID-19. Until a vaccine is ready, widespread testing is one of the strategies to “flatten the curve” or slow down the spread. As a result of this, some control measures, namely contact tracing, social distancing or physical distancing [3], large-scale screening and testing, and “lockdown” have been adopted to mitigate its effect on society. Contact tracing is very vital in an outbreak for public health to help possible early diagnosis and getting care to infected persons, as the information is significant for public health authorities to contain the pandemic. Moreover, if the pandemic spread outpaces the contact tracing effort, cases may begin to emerge outside the chain of transmission. As the number of COVID-19 cases continues to rise, health workers and health facilities are increasingly overwhelmed. The high numbers have made it difficult if not impossible for the healthcare sectors in developing and developed economies to cope with the upsurge.

The lack of diagnostic equipment/kits has also become a barrier to the effective identification and management of infected persons to curb further infections. Accordingly, the need for very sensitive diagnostic tools by healthcare workers to engender quicker identification of potential cases of COVID-19 remains imperative.

Pandemics such as COVID-19 affect micro- and macro-economic institutional settings, particularly as they concern reprioritization and reallocation of public sector expenditure towards the healthcare sector, drops in household income bringing about changes in consumption patterns and increased production costs due to the uncertain nature of the business environment [4]. African countries are not immune from the negative impacts of the pandemic on economic performance. For example, in the aviation subsector of the transportation sector, Ozili [5] reported on the drastic drop in revenue for African airlines, culminating in a loss of an estimated USD 400 Million Furthermore, the author observes the slump witnessed in the shares index of listed companies across various stock exchange platforms within the continent—Johannesburg, Nairobi and Nigerian stock exchange platforms—an effect attributed to the extant linkages between these firms and the Chinese economy. Further, the tourism sector in South Africa and Kenya has been gravely affected due to the closure of the national borders with 80% and 55% falls in tourist visits, respectively, in these countries [5]. Moreover, the rapid increase in the number of newly confirmed COVID-19 presents a worrying situation for governments and public health workers. These worries are predicated on the possibility of the number of infected cases overwhelming the healthcare infrastructure, especially the number of available beds, ventilators and personal protection equipment (PPE), as well as medical personnel required to attend to the incidence of COVID-19. This is already a reality in developed countries with a reputation for having excellent healthcare infrastructure and highly skilled medical personnel [6,7].

It is projected that the state of healthcare infrastructure in developing countries like South Africa exposes such healthcare systems therein to being easily overwhelmed if an exponential increase in the number of COVID-19 cases is experienced [5,8,9]. This understanding and the need to manage the rate of infections across the globe culminated in the lockdown of various economies, thereby resulting in movement restrictions, social distancing, deployment of rapid testing platforms and contact tracing. Based on the foregoing, the importance of identifying and isolating infected cases to prevent rapid transmission or to flatten the transmission curve remains imperative [10]. Therefore, the diagnosis of cases in a manner that is commensurate to the rate of disease transmission between infested persons remains critical to containing the spread and reducing the death rate. As countries strive to find quick diagnostic test kits, some developing nations such as Senegal—a West African nation—have developed a quick self-diagnostic test kit that uses human saliva or blood deposited onto a device, to automatically detect any symptoms of COVID-19 as quickly as possible for the needed health intervention [11]. While those with active infection use a saliva swab, those with previously undetected cases would use an “at-home” finger prick test to check for coronavirus antibodies. However, the availability of test kits for mass screening remains a challenge. Other African countries, such as South Africa, have adopted mobile text messages and the WhatsApp platform to assist in predictive modelling and planning an appropriate response for reported cases.

While researchers explore ways to improve on the accuracy of predicting COVID-19 cases, which the current conventional artificial intelligence models are challenged with, it is needful to also explore the potential of computational models to contribute in this time of crisis. In this regard, the performance of current models is the criteria for their use in detecting and predicting cases of COVID-19. In this study, we reviewed nature-inspired, big data and AI-based technologies as veritable models for the detection and prediction of COVID-19. The study is motivated by the fact that in terms of accuracy, machine learning performance can be misleadingly high in a classification problem when a class is under-represented or a dataset is limited [12]. The contribution of this paper is as follows: (1) to identify the current AI-based interventions for the screening and diagnosis of COVID-19 pandemic cases and its use in contact tracing and case detection; (2) to explore the use of big data for contact tracing and case detection; and (3) to ascertain the possible combination of AI models and big data analytics with nature-inspired computing (NIC) to enhance the accuracy of the detection of COVID-19 cases.

## 2. Background

As mentioned earlier in the introduction, contact tracing is one of the control measures adopted to reduce the spread of COVID-19. It has been described as a public health response strategy for tackling infectious disease outbreaks, “especially in the early stages of an outbreak when specific treatments are limited” [13]. The advantage of contact tracing lies in its ability to identify potentially infected individuals before severe symptoms emerge, and “if conducted sufficiently quickly can prevent onward transmission from the secondary cases” [13]. The routine contact tracing method starts with an interview of a confirmed case to find persons they might have had face-to-face contact with or where there may have been a spread of COVID-19 through an instance of droplets being sprayed. Such an interview gives a detailed history of where the person/patient has visited/travelled to, who they may have sat with, where they may have had a meal and where they may have slept, so this historical information is useful in identifying all possible contacts [14]. The information on travel or movement can be considered as a human mobility issue.

Human mobility tools [15] which provide location tracing services are some potential ways to collect self-reported data and for tracing infection within rural settings where network coverage and internet infrastructure are poor. The use of Google Location History (GLH) for instance, provides a way of collecting human mobility data [16]; however, the underutilization of GLH data by mobile apps is one of the challenges hindering the availability of human mobility data to understand human movement [16]. Additionally, there is a lack of interoperable apps to ensure contact tracing of people as they travel between different countries using different tools.

The contact tracing method has been applied for disease outbreak detection such as tuberculosis [17], Ebola outbreak in 2014 [18], severe acute respiratory syndrome (SARS) outbreak in 2003 [19], Middle East respiratory syndrome (MERS) outbreak in 2012 in the Middle East Saudi Arabian Peninsula region and MERS outbreak in 2015 in South Korea. Data on location history of where a patient who is confirmed may have gone to sit or eat can be collected from train stations, airports, restaurants, hotels, shopping malls, etc. The data collected from these different sources are either structured or unstructured, thereby implying the need for such data to be pre-processed. Unstructured or structured data are one of the characteristics of big data and thus contact tracing can be considered as a big data problem. By using big data analytics tools, contact tracing information can quickly be processed to find the “hot-spot” of COVID-19 cases, for the needed control measure, e.g., lockdown. In COVID-19, the type of data that has been used for predicting cases include the following: the use of clinical data, the use of online data and biomedical data [20]. The most effective way to prevent the infection and save the lives of millions of people is by breaking the chains of transmission and this is achieved by testing and isolation [11].

The adoption of fourth industrial revolution (4IR) technologies, particularly artificial intelligence (AI) and big data, is expected to make significant contributions towards facilitating contact tracing [21]. The emergence of AI-based technologies that have been deployed for medical image acquisition and analysis of COVID-19 pandemic cases will help to automate the scanning and detection procedures especially as it concerns the reduction in the contact between patients and frontline hospital workers [22]. Further, computer-aided diagnosis tools are needed to provide the accurate screening and detection of cases.

## 3. Related Works

### 3.1. Big Data Analytics

Big data is a term that arose out of the need to analyze large volumes of unstructured data generated every second from different data sources [23]. Mostly, traditional analytics tools are not adapted to process such unstructured data in order to find some insights [24]. Big data can be characterized by speed, a large amount of data and a variety of data sources (e.g., heterogeneous sources). The challenge with big data is how to conduct the analysis as quickly as possible with some degree of accuracy. Currently, big data is spawning new tools that combine machine learning models for data analysis. The benefit of big data analytics is the real-time monitoring and forecasting of events. Big data in healthcare refers to the high volumes of health-related data from different sources including medical imaging, pharmaceutical data, electronic health records and many more. Pham, Nguyen [21] referred to big data in the context of COVID-19 as the patient care data, namely “physician notes, X-Ray reports, case history, list of doctors and nurses, and information of outbreak areas”. Within the context of contact tracing, big data could be referred to as the contact information collected from different sources, hotels, airports, restaurants, etc., that can help with tracing any person who has been being in close contact with a confirmed case of COVID-19. Although there is no definition for contact tracing big data analytics, we define it as the collection and analysis of social historical contact information from diverse sources, that are changing with time and growing quickly.

The characteristics of big data include the following:

- Volume is the amount/size of data that must be processed. The size of this big amount of data ranges from thousands of terabytes to petabytes and exabytes [23];
- Veracity is referred to in this research as the accuracy of results from a processing system [25];
- Value relates to what the user will gain or the benefit from the analysis results;
- Variety is the different kinds of data that are generated, namely structured or unstructured data (e.g., email messages, transcripts of call center interactions, posts from blogs and social media sites; images; audio; video files; and machine data such as log files from websites, servers, networks and applications from mobile systems) [26];
- Velocity is how fast data are created, processed and updated and how quickly the user of information needs results from the processing system [27].

These characteristics of big data apply to different problem domains including the current pandemic of coronavirus.

#### 3.1.1. Contact Tracing with Big Data Analytics

Contact tracing through big data is a very important domain that can assist with disease outbreaks, using different sources of data, e.g., posts with meta-data and tags used on social media, passenger lists, smartcards to metro, logs of a vehicle that people travel with and the use of credit card are all valuable sources of data. By determining features of importance from location meta-data/tag on social media to validate that a person was at a certain location at a certain time, although not accurate, can provide a tracking model that could trace people even if they do not have tracking equipment or mobile phones with them.

Keeling, Hollingsworth [13] proposed a contact tracing model to estimate the transmission rate of infection. This model identified potentially infected individuals before severe symptoms emerged; however, if not conducted “sufficiently quickly”, it has its repercussion on society. It was suggested that one of the most notable features of human social contacts is the huge variability in the number and degree of contacts in both the number of secondary cases and the number of individuals that match the “contact-tracing” definition. Heterogeneity of social encounters to determine the transmission rate, which is stochastic, hinges on reliable data which are often limited [27]. Big data can ensure the availability of the needed information for a quick determination of the rate of transmission on a large scale. However, the data architecture is essential to ensure data merging, sharing and its analysis [28] within the big data environment. The data architecture can enhance the real-time monitoring of different public health surveillance systems. When big data is collected from social networks, it can be reconstructed early to determine an outbreak of disease. Mostly, in the early stage of an outbreak, non-classical datasets and data streams can inform how the data architecture should be design and implemented for an effective outbreak determination [29].

#### 3.1.2. Case Detection with Big Data Analytics

Qin, Sun [30] used social media search indexes (SMSI) to detect new cases either suspected or confirmed. SMSI lists the keywords that appear on a social media site to predict new COVID-19 cases including clinical symptoms such as cough, pneumonia, fever and chest distress. Movement of people has been identified as a keyway of spreading infection, in this regard, posts on social media can help to identify any information relating to the perceived risk of contracting the infection. However, such posts might not be accurate and reliable, which remains one of the challenging issues in social media big data analytics.

The advantage of using keywords as an approach is that health authorities can outline appropriate responses to create the needed alert mechanism. Unfortunately, the COVID-19 pandemic has a widespread geographical dimension [31]. Big data provides a suitable means to map and merge different data sources to help understand COVID-19 outbreak tracking. This can also help to know the virus structure and treatment because big data platforms can be equipped with complex modeling tools that help to build complex simulation models using the COVID-19 data stream [21]. Thus, text mining algorithms are imperative. However, the volume of data and how quickly they must be processed calls for the use of AI-based intervention.

The summary of the big data analytics platforms and their application domains is presented in Table 1.

Stream data processing requires real-time analytics and considering the geographical dimension of recorded cases of COVID-19, big data platforms can enable data collection and storage in scattered datasets [32]. Storm, Splunk and Apache are real-time stream processing platforms with low latency response. Storm uses a mapping strategy to interconnect data from different data sources for real-time processing. The advantage is a stable distributed environment that it provides using “master” and “work node” to allow delegation of the parallel processing mechanism to its analytics framework [33]. S4 is a general-purpose, distributed environment that has a pluggable computing framework for easy development of big data processing applications. Kafka is an open-source framework that is used for the collection of logs. Flink is a dispersed processing platform for stream processing where the processing of data is performed as “cyclic data flow with several iterations”. By using the set of iterations, it reduces the data computation time. Apache Spark uses machine learning algorithms for clustering and classification. With Spark, data are mapped, reduced and filtered by passing information to the spark runtime framework. Finally, the hybrid processing is based on Lambda architecture, which is a data processing architecture for massive data processing. The architecture supports both batch and stream processing of data, and the results of processing are merged by combining batches and the real-time view of batch [34].

### 3.2. Artificial Intelligence Models

Artificial intelligence (AI) focuses on creating machines referred to as an intelligent agent that can engage on behaviors that humans consider intelligent. The term ‘‘intelligent agents,’’ refers to any autonomous device capable of perceiving its surroundings and takes actions that maximize its chance of success at some goal [35]. By operating autonomously, it needs to handle effectively large heterogeneous data sources and process data within a limited duration [35]. In so doing, several of these agents can help to create an inter- and intralayer operability of systems [36], which are self-learning systems capable of handling any prediction-related task [35]. The learning and training of AI systems before actual deployment into their real-world environment requires training with a dataset which is very costly because the model learns from the entire dataset, thereby producing output based on data in the memory (e.g., over-fitting). Among many branches of AI, machine learning (ML) and deep learning (DL) are two important approaches. Generally, ML refers to the ability to learn and extract meaningful patterns from the data, and the performance of ML-based algorithms and systems is heavily dependent on the representative features. The architectures of ML are simple and consist of a single layer, which transforms the raw input signals into a problem-specific feature space. Unfortunately, these architectures are effective only in solving well-constrained or simple problems. However, they have limitations in dealing with unstructured or complex problems [37]. Alternatively, DL is a class of machine learning algorithms that exploits several layers of representation to find a complex and inherent relationship in data [38]. Learning by representation allows a machine to receive raw data and to automatically find the vector representations needed for the detection or classification of a task.

Deep learning is making a major advance at discovering intricate structures in high-dimensional data which performs better at predicting the activity of potential drug molecules [39], the effects of mutations in non-coding DNA on gene expression and disease [40]. Deep learning has improved the state-of-the-art in many application domains such as object detection, drug discovery and genomics [41]. This improvement is enabled by using a back-propagation algorithm to learn the internal parameters required for the computation of a new representation in its layer from previous layers. Deep learning has also been applied to visual tracking [42] and object identification in medical images analysis [35,43].

#### 3.2.1. Case Detection with Artificial Intelligence Models

AI and ML have been adopted in the screening of populations of persons to assess the risk of infection [44]. For instance, such AI-powered temperature screening was deployed in public locations in the COVID-19 pandemic by China. Temperature screening has helped to detect symptoms and isolate persons who are suspected to be infected. Recently, thermal cameras have been used to provide thermal imaging for body temperature for COVID-19 case detection, which helps to quickly and accurately identify people who have “elevated body temperatures”, which is one of the key symptoms of COVID-19 [45]. Additionally, an AI-powered smartphone app was developed to track the geographical spread of COVID-19. Such apps are aimed at predicting which population and communities are the most susceptible; to enable real-time information dissemination from the medical health providers; and to notify individuals of potential infection hotspots in real-time so as to avoid such areas [44].

Other AI-based technologies include BlueDot to find emerging risk of infection [46], chatbots used as virtual assistants to provide information on the virus; and diagnostic robots used to diagnose disease.

With the case of the 2015 Zika virus epidemic, a dynamic neural network was developed to predict its spread [47]. Predictive models have been developed such as the seasonal auto-regressive integrated moving average (SARIMA) model to predict influenza changes [48], SARIMA and a support vector regression model to predict the incidence of hand-foot-mouth disease (HFMD) [49] and many more. However, the challenge with the adoption of predictive models for any pandemic is the lack of historical data [42]. Big data models and AI models can be leveraged upon to train models and develop robust predictive models. Incorporating data from countries that were hit earlier is significant in aiding the development of machine learning models as more data are retroactively posted from medical sites [50]. By adopting AI models, the spread of infection can quickly be identified for the needed intervention. AI can ensure early warning and alert, tracking and prediction, diagnosis and prognosis, treatments and cures, and social control. One of the possible ways to find patient infection to COVID-19 is by analysing the chest X-ray images and because of the large number of patients in hospitals it is time-consuming and difficult to examine a high number of X-ray images [51]. A deep convolutional network model was adopted to classify X-ray images into normal, pneumonia and COVID-19.

In the study by Rahimzadeh and Attar [51], a training technique that combines Xception and the RasNet50V2 network to improve the accuracy of detecting COVID-19 from 11,302 images yielded an accuracy of 99.56% and the overall accuracy for all classes was 91.4%.

Currently, an automatic framework to detect COVID-19, using a dataset of 4356 chest computed tomography (CT) exams from 3322 patients, was developed using a deep learning convolutional network model (COVNet) and its diagnostic performance was 0.96 [52]. The robustness of the model was tested on community acquired pneumonia (CAP) and non-pneumonia CT exams and its diagnostic performance was 0.95. Xu, Jiang [53] proposed a deep learning model for early screening to detect COVID-19 patients which resulted in an accuracy of 86.7%. A deep learning convolutional neural network model was also used to predict whether a person is affected with COVID-19 by analysing CT scans which achieved an F1 of 0.85 when tested on a dataset containing 275 CT scans [54]. Upon extraction of the features of COVID-19 on chest X-ray using a deep convolutional neural network, VanBerlo and Ross [12] went on to apply the local interpretable model-agnostic explanations (LIME) algorithm [55] to help with the explanation of the features generated by the model for healthcare workers. Narin, Kaya [56] developed an automatic detection system based on a convolutional neural network with an accuracy of 98%, as an alternative and quick diagnosis option to prevent the wide spread of COVID-19 due to the limited number of COVID-19 test kits in hospitals as the result of the high number of cases recorded.

It is no longer news that the large number of recorded cases has overwhelmed the extant health sector capacity. Accordingly, Rahmatizadeh, Valizadeh-Haghi [57] proposed an AI system that automatically analyzed CT images to detect features of COVID-19 pneumonia. The robustness of this model was tested with 1136 training cases with 723 positives for COVID-19, which was collected from five hospitals and the results were promising. An AI-based management model for the critical care of COVID-19 patients in ICU was proposed by Rahmatizadeh, Valizadeh-Haghi [57]. Vaishya, Javaid [58] compared non-AI-based treatment systems with AI-based systems and suggested that an AI-based system reduces the time and processes in the treatment of a patient and can also help with the control of disease. Additionally, the AI-based system can identify the level of infection in a location which can assist with contact tracing [58].

ELGhamrawy [59] presented a deep learning-based CNN model (Artificial Intelligence-inspired Model for COVID-19 Diagnosis and Prediction for Patient Response to Treatment—AIMDP), for COVID-19 diagnosis and prediction of patient response. The diagnosis module detects patients with COVID-19 by feature selection and classification, and the prediction module predicts the patient’s response to COVID-19 treatment.

Chae, Kwon [60] indicated the difficulty to act against infectious disease as one of the challenges in predicting infection. This is attributed to the delay in receiving infection reports and the likelihood of having missing reports on infection, which leads to unknown trends of infectious diseases. Thus, providing an optimized prediction parameter through a deep learning algorithm was used on a big data environment particularly on social media data. A simulation of the performance of the prediction of deep learning models (that is the deep neural network and long-short term memory) was compared with the traditional autoregressive integrated moving average (ARIMA) to predict three infectious diseases in advance, a week ahead [60]. The empirical results alluded that deep learning models are accurate in predicting infectious disease spread. Infectious diseases have led to premature death worldwide [28] and it is imperative to explore the use of AI models for the timely prediction and tracing of factors that affect the process of infection and its transmission.

Anuradha and Gupta [61] suggested the use of a long short-term memory model for the prediction of the number of COVID-19 cases in India and also to find how effective the social distancing measures were on the spread of the pandemic.

#### 3.2.2. Contact Tracing with Artificial Intelligence Models

Artificial intelligence technologies are used to track and identify the spread of the COVID-19 pandemic. The smartphone technologies that use AI have been deployed to trace a person’s location and also to scan public space for potentially affected persons through the use of fever detecting infra-red cameras, computer vision surveillance and facial recognition systems. Countries such as South Korea and Singapore have implemented a contact tracing app for COVID-19. In the case of South Korea, GPS phone tracking, credit card history, surveillance video recording and personal interviews with patients were used for the contact tracing of COVID-19 infection. Additionally, both countries have implemented contact tracing using location information on smartphone apps. Meanwhile, in Singapore, TraceTogether is a mobile application that enables community-driven contact tracing where devices exchange their proximity information and duration through Bluetooth signal whenever their app detects another phone with the app installed on it. With such a community-driven app, “no location information” is the collection, however, the individual is legally bonded to give any information stored by their phone that might assist with contact tracing. As COVID-19 has spread very quickly, contact tracing technologies can help to speed up the process of tracing the infection. Further, AI models have been used to monitor crowd information to enforce a physical distancing guide. The challenge with AI-based technologies in terms of monitoring crowd information raises some privacy issues which should be addressed by the developers of AI technology.

Table 2 shows a summary of deep learning algorithms and their application domains.

The CNN model is the most widely used model for image detection and classification as evident from the literature reviews. This can be attributed to its robustness in terms of detection of cases and their performance (that is, accuracy). Other deep learning models that are sparingly used include long-short term memory (LSTM), recurrent neural network (RNN) and many more. The ensembling model that combines the predictive capabilities of these models is proposed because it helps to assign equal weights to combined models [66]. For instance, LSTM and RNN have been applied to predict the life expectancy from electronic medical records [67]. Additionally, an LSTM and RNN model was used to predict the epidemic of COVID-19 [68]. The AI applications’ interventions for the COVID-19 pandemic include the early “detection and diagnosis of the infection”, prevention of disease, monitoring the treatment, contact tracing of the individuals, drugs and vaccines development, reducing the workload of healthcare workers and projections of cases and mortality [58].

The outbreak of COVID-19 has led to the development of several deep learning models for diagnosis, prediction and treatment. Hanumanthu [69] suggested that most of the reviews of the literature on COVID-19 prediction/diagnosis were conducted using deep learning models followed using machine learning models, then mathematical and statistical models for the prediction of COVID-19. Deep learning was applied for feature extraction without human input and has shown good performance. Additionally, its ability to handle complex and multi-modal data are among the reason for its use for COVID-19 cases. In Hanumanthu [69], deep learning approaches that were used in different problem domains showed that the accuracy ranging from a minimum of 85% and 98.3%. Similarly, Kumar, Gupta [62] identified the accuracy of AI-based technologies as ranging from 79.3% to 95%. The reason for variation in accuracy is due to data availability on factors which are not limited to age and chronic disease, and different models’ parameters. Hence, aligning the source of data to a deep learning model is one of the key issues that need to be solved aside the quality of the real-time datasets.

### 3.3. Nature-Inspired Computing Models

Nature-inspired computing (NIC) is an interdisciplinary domain that combines the branch of computing science with other knowledge disciplines from sciences, e.g., physics, chemistry, biology, mathematics and engineering, to allow for the development of new computational tools such as algorithms and hardware [70]. NIC intelligence is an optimization technique based on the behavior of living organisms. An NIC model seeks to achieve an objective within a set of constraints to be satisfied and the optimal solution is measured by a performance index known as objective function [70]. The nature-inspired algorithm can explore and exploit the search space using the naturally endowed random hunting skills of animals to find a global optimal solution [71]. NIC algorithms are mainly categorized into evolutionary algorithms and swarm intelligence-based algorithms. Evolutionary algorithms are based on the evolutionary behavior of natural systems, e.g., genetic algorithm (GA), whereas swarm intelligence mimics the collective behavior of natural swarms, e.g., the ant colony algorithm (ACO) [72], and the wolf [73], ANT [74], dung beetle [75], particle swarm optimization (PSO) [76,77] and BAT [78] algorithms, plus many more. Table 3 shows the nature-inspired algorithms and application domains.

#### NIC for Contact Tracing and Case Detection

The prominence of NIC across several application domains shows its potential in solving many problems. The NIC models have been applied to the diagnosis and prediction of COVID-19 [59], and they have been used for X-ray image segmentation to identify features of COVID-19 [91]. As early detection and treatment of COVID-19 is very needful, the review of the current literature suggests that nature-inspired computing models have not been explored in the contact tracing algorithms for the COVID-19 pandemic. Meanwhile, while nature-inspired computing models have been applied in domains such as car tracking algorithms [92], similar studies for the COVID-19 pandemic should be given the needed attention by the research community.

## 4. Shortcomings of Current Methods

Currently, the accuracy of automated screening and diagnosis of the COVID-19 pandemic case poses a challenge for AI-based technology interventions such as deep learning CNN models. Since AI algorithms suffer performance issues, by applying nature-inspired computing models, the performance could be improved. In so doing, it guarantees the accuracy of detection that can ensure the effective treatment and diagnosis of COVID-19 cases.

Besides, the tracing of contacts on a wider scale is heterogeneous in terms of social encounters [13], and as contacts add up fast, hence requiring more outreach, work to trace every contact is needed which can be very costly [14]. Table 4 shows the shortcomings of AI-based technologies, big data analytics and capabilities of NIC at resolving the shortcomings.

## 5. Future Research Direction

The recent outbreak of COVID-19 has created new research dimensions and the application of technologies. Nature-inspired computing systems have many attractive properties such as self-organization, ease of implementation and being dynamic and flexibile, which can be applied to enhance the performance of the current screening, diagnosis and treatment of COVID-19 cases.

Besides, the complexity of the COVID-19 pandemic presents multiple areas of exploration. Some examples include tracing the mutation of the virus and identifying the currently lesser-known effects of COVID such as cognitive impairment, organ damage and mental health issues amongst its patients. The pandemic’s effects on the real word also provide various contexts of investigation such as the effect on the economy in its various aspects, the growth of telework and online education and crisis management.

The task of exploring these areas is quite daunting but the use of combinations of selected NIC algorithms may assist in the detection of infection. The ACO algorithm may be used to identify outliers among infected patients such as patients in lesser health risk categories dying. The IACO may be used to quickly obtain required information from diverse sources to treat an infected patient more effectively. The ABC algorithm may identify “hot-spots” of a given phenomenon, such as the spread of a rare mutation occurring. The SFL algorithm may be used, in the real world, to filter out irrelevant variables of an event and to focus on relevant factors only. The BFO algorithm may also be used to retrieve the relevant information from the medical dataset such as CT images. The CSO algorithm may be applied for text classification in big data contact tracing to select features of a positive case of infection and match with potential cases.

Currently, we identified a blend of CNN and the whale optimization algorithm for COVID-19 diagnosis and prediction of the patient’s response to treatment [59]. The whale optimization algorithm was applied to select relevant patients’ features, which demonstrates very promising results. The review of nature-inspired computing indicates the wider application domain for real-life problems. Based on the review of AI, nature-inspired computing models have not been fully explored for the early screening and detection of COVID-19. Models such as WOA and other similar AI-nature-inspired computing-based models are significant to improve the accuracy of detecting COVID-19 cases.

The contact tracing big data analytics model is a predictive model that combines the capabilities of AI-based models and NIC in tracing different contacts to select relevant features that are needed in tracing contact locations. The advantage of the contact tracing effort is that it helps to recommend self-quarantine and to contain the widespread infection. By utilizing NIC, we can fine-tune different random parameters for an AI-based model to select the relevant features. Further, NIC can be applied to optimize the rate of transmission at the contact tracing phase.

The flock by leader algorithm can also be explored for proximity detection to trace contacts due to its hierarchical representative nature such that leaders, in this case, the COVID-19 infected person, can be traced to their followers, that is those who came in contact with an infected person, within a virtual space [90]. Similarly, this algorithm can be applied to data clustering by grouping related features of COVID-19 from non-COVID-19 cases. Practically, the implementation of the detection of cases and contact tracing approaches requires data on medical images and textual data to be collected from different sources, hence its architecture provides issues that need to be addressed for the successful detection of COVID-19 cases.

## 6. Conclusions

In this study, we provided a review of models of nature-inspired computing, artificial intelligence and big data for contact tracing. The review sheds light on the prospects of these models for the screening and detection of COVID-19. The current AI-based technology interventions for screening, diagnosing and predicting COVID-19 cases are challenged in terms of the accuracy of classification for effective prediction and diagnosis. The application of a combined nature-inspired and artificial intelligence model can aid in the accuracy of screening to detect relevant details only amidst a multitude of interacting factors in the real world. We propose the use of nature-inspired computing models to identify pertinent features and reduce remaining features, combined with the use of the artificial intelligence-based CNN model to increase the feature identification accuracy of COVID-19′s diagnosis and patients’ responses to a given treatment. Additionally, big data analytics tools can be adopted in the contact tracing of the COVID-19 case to identify “hot-spots”, and to alert people. Subsequently, we also propose a contact tracing big data predictive analytics model.

## Figures and Tables

**Table 1:** Summary of big data analytics.

Real-Time Big Data Analytics Platform	Application Domains
Storm	For data stream processing in real-time that focuses on message processing [32].
S4 and Kafka	For distributed stream processing inspired by the MapReduce model
Flink	Supports batch processing
Apache Spark	For stream processing that can process large volumes of data in memory with limited time.
Hybrid processing	Supports both batch and stream processing

**Table 2:** Summary of artificial intelligence (AI) algorithms.

Artificial Intelligence Algorithms	Application Domains
CNN	For image detection and classification, Early detection of COVID-19 using radiology images [62]
RNN	For predicting patients’ future health information [63]
LSTM	Performs diagnosis classification from the clinical measurement of the patient in a pediatric intensive care unit [64], prediction of future medical outcome [65], drug discovery [20]

**Table 3:** Summary of nature-inspired algorithms.

Nature-Inspired Algorithms	Application Domains
GA	Bandwidth utilization, computational resources and data dependencies [79]. GA algorithm was applied to decoupled data and computational services on the cloud.
Simulated Annealing (SA) and Whale Optimization Algorithm (WOA)	SA algorithm-based big data optimization technique, which uses WOA to design different feature selection [80]. SA algorithm helps to improve the classification accuracy and selects the most useful attributes for classification tasks.
PSO	Time series prediction, remote sensing image registration [81].
SA algorithm based on feature selection	SAFS technique for big data learning and computer vision. SAFS algorithm removes variables and tightens a sparse constraint, to reduce the problem size that makes it mainly fit for big data learning [82].
Artificial Bee Colony (ABC)	ABC algorithm-based clustering approach for big data, which identifies the best cluster and performs the optimization for different dataset sizes. ABC algorithm minimizes the execution time and improves the accuracy of clustering when implemented on a map/reduce-based Hadoop environment. ABC algorithm has been applied in training neural networks for pattern recognition [83].
Firefly Swarm Optimization (FSO)	FSO algorithm-based hybrid (FSOH) approach for big data optimization, which focused on six multi-objective problems to reduce the execution cost but it has high computational time complexity [84]. Heart disease prediction, image processing (segmentation of brain tissues, multilevel color image thresholding), clustering and classification (protein complex identification, hyper-spectral image classification [81].
Grey Wolf Optimization algorithm (GWO)	Feature selection, community detection, iris recognition [81].
Cat Swarm Optimization (CSO)	CSO algorithm-based approach for big data classification to select features in a text classification experiment for big data [85]. CSO algorithm uses the term frequency-inverse document frequency to improve the accuracy of feature selection.
Ant Colony Optimization (ACO)	For mobile big data to select optimal features to resolve decisions, which aids to manage big data of social networks (tweets and posts) effectively [86]. Predictive control for nonlinear processes, anomaly detection, treating missing values in big data sets, medical image de-noising, hyper-spectral image classification [81].
Improved ACO algorithm (IACO)	Big data analytical approach for management of medical data such as patient data, operation data, which helps doctors to retrieve the required data in little time [87].
Shuffled Frog Leaping (SFL)	Selection of the feature in high-dimensional biomedical data. SFL algorithm maximizes the predictive accuracy by exploring the space of possible subsets to obtain the set of features and reduces the irrelevant features [88].
Bacterial Foraging Optimization (BFO) algorithm	Classify the informative and affective content from the medical weblogs. The “MAYO” clinic data were used to evaluate the accuracy to retrieve the relevant information from the medical dataset [89].
Kestrel-based Search Algorithm (KSA)	KSA was applied as a parameter tuning algorithm to improve on the accuracy of feature selection in high-dimensional bioinformatics datasets [76].
Lion Optimization Algorithm (LOA) Lion cooperation characteristic	Data clustering, extracting liver from the abdominal CT images [81].
Whale Optimization Algorithm	Feature selection and currently proposed for diagnosing and predicting COVID-19 cases [16].
Flock by leader	For local proximity in an artificial virtual space [90], data clustering.

**Table 4:** Shortcomings of AI-based technologies and capabilities of nature-inspired computing (NIC).

Shortcomings of Big Data Analytics	Shortcomings of AI-Based Technologies	Capabilities of NIC to Resolve Shortcomings
Determining the accuracy and reliability of social media posts.	Accuracy of classification of features is by backward propagation methods that suffer from over-fitting and under-fitting [93].	Uses randomized parameters to find the most or near-optimal solution that maximizes the classification accuracy.
The architecture for data sharing and merging remains one of the shortcomings considering the widespread geographical dimension for quick case detection and tracing of contacts.	Once the network learns one set of weight, any new learning causes catastrophic forgetting [94]	It applies randomization to learn new parameters to optimize learning.
	Back-propagation can be optimized locally but it fails globally which affects the accuracy of classification.	Ability to exploit local search and find the most optimal global result [76].
